# The Critical Role of Vitamin D Supplementation for Skeletal and Neurodevelopmental Outcomes in Preterm Neonates

**DOI:** 10.3390/nu17081381

**Published:** 2025-04-19

**Authors:** Roberta Leonardi, Carmine Mattia, Nunzia Decembrino, Agata Polizzi, Martino Ruggieri, Pasqua Betta

**Affiliations:** 1 Postgraduate Training Programme in Pediatrics, Department of Clinical and Experimental Medicine, University of Catania, 95123 Catania, Italy; 2 Neonatal Intensive Care Unit, AOU Policlinico G. Rodolico San Marco, 95123 Catania, Italy; lorettamattia@hotmail.com (C.M.); n.decembrino@policlinico.unict.it (N.D.); mlbetta@yahoo.it (P.B.); 3 Unit of Pediatric Clinic, Department of Clinical and Experimental Medicine, University of Catania, 95123 Catania, Italy; agata.polizzi1@unict.it

**Keywords:** premature infants, metabolic bone disease of prematurity, vitamin D supplementation, neonatal neurology

## Abstract

**Background/Objectives:** Metabolic bone disease of prematurity (MBDP) is a multifactorial disorder resulting from disrupted transplacental mineral transfer and postnatal nutritional deficits, particularly affecting preterm neonates born before 32 weeks of gestation or weighing under 1500 g. Although substantial research has focused on skeletal outcomes, few studies have explored the association between MBDP and neonatal neurological impairment. This narrative review is the first to integrate the pathophysiological mechanisms, diagnostic methods, and preventive strategies for MBDP, while simultaneously investigating its potential impact on neurodevelopment. **Methods:** A narrative review of recent peer-reviewed studies, systematic reviews, and clinical trials was performed focusing on biochemical markers (alkaline phosphatase, FGF23, calcium, and phosphorus), emerging tools such as bioelectrical impedance analysis (BIA), and the effects of early nutritional interventions on both skeletal and neurodevelopmental outcomes in preterm infants (n = seven included articles). **Results:** Early elevations in ALP, particularly when combined with low serum phosphorus and FGF23 levels, provide sensitive markers for identifying MBDP. Furthermore, insufficient vitamin D levels during gestation and in the neonatal period have been associated with increased risks of seizures, hypotonia, and developmental delays. Studies suggest that enhanced vitamin D supplementation in preterm infants (up to 800 IU/day) may improve mineral absorption and bone formation and confer neuroprotective benefits through anti-inflammatory and antioxidant mechanisms. **Conclusions:** This is the first review on the neurological implications of biochemical actors of MBDP. As a result, diagnostic and therapeutic strategies, including vitamin D supplementation, can improve bone and neurodevelopmental outcomes. Future prospective studies are required to standardize diagnostic criteria and optimize therapeutic regimens for enhanced long-term benefits.

## 1. Introduction

Metabolic bone disease of prematurity (MBDP) is defined by tissue reduction and postnatal bone demineralization with typical clinical, biochemical, and radiologic features presenting at 4–8 weeks of postnatal life, possibly leading to growth deficits or fractures in the most severe cases [1,2].

Preterm infants, particularly those born with a gestational age below 32 weeks or a birth weight under 1500 g, are at a heightened risk of developing metabolic bone disease of prematurity (MBDP) due to an interruption in the critical period of mineral accretion typically occurring in the third trimester [3]. The abrupt cessation of transplacental calcium and phosphorus transfer, compounded by challenges such as gastrointestinal immaturity and prolonged parenteral nutrition, leads to insufficient bone mineral content [4]. Around 40% of newborns with a very low birth weight and 50% of newborns with an extremely low birth weight manifest MBDP, with an incidence estimated between 6.7 and 56.3% [5,6].

MBDP can manifest as rickets-like changes, pathological fractures, and long-term deficits in bone growth [7]. Fractures have been described in up to 33.8% of cases (including rib fractures) and skeletal abnormalities such as rickets, poor skeletal support, and widened anterior fontanels [1,8]. Other typical characteristics are as follows: increased respiratory work related to rib fractures, frontal bossing, and the enlargement of brain suture size [9].

Recent studies have increasingly focused on early interventions and diagnostic methods to mitigate the morbidity associated with MBDP. However, there are no specific diagnostic methods for MBDP, leading to late diagnosis or misdiagnosis; also, there is no universal consensus regarding treatment in neonatal intensive care unit (NICU) settings [9,10]. Additionally, the long-term outcomes of MBDP, specifically neurological outcomes, are still unknown [11]. Recent systematic reviews and meta-analyses have begun to shed light on the broader implications of disturbed vitamin D homeostasis during the perinatal period. García-Serna and Morales (2020) [12] demonstrated that higher prenatal levels of 25-hydroxyvitamin D (25(OH)D) are positively correlated with improved cognitive outcomes and a reduced risk of attention deficit hyperactivity disorder (ADHD) and autism-related traits in offspring. In particular, lower prenatal vitamin D exposure correlates with a higher risk of ADHD and impaired cognitive development. This evidence underscores that optimizing vitamin D status during pregnancy may have critical neurodevelopmental benefits [12]. In parallel, a recent retrospective cohort study by Chen et al. (2024) focusing on very-low-birth-weight infants has found that those diagnosed with MBD exhibit significantly lower neurodevelopmental outcomes across cognitive, motor, and language domains at 2 years of corrected age compared to their peers without MBD [13]. These findings suggest that the skeletal deficits associated with MBDP may extend their impact to critical aspects of neurodevelopment, highlighting an urgent need for integrated management strategies that address both bone health and neurodevelopment in this population [13].

This narrative review aims to elucidate an in-depth examination of the pathophysiological mechanisms underpinning MBDP, the diagnostic methodologies, including emerging tools such as bioelectrical impedance analysis (BIA), and preventive supplementation strategies, particularly with vitamin D. Emphasis is placed on the dual benefits of vitamin D supplementation, not only in bolstering bone mineralization, but also in mitigating adverse neurodevelopmental outcomes. By integrating current evidence on both the skeletal and neurological dimensions of MBDP, this review aims to address a critical gap in the literature and propose practical, evidence-based recommendations for the early management of MBDP in preterm infants.

## 2. Organization of the Review

To guide the reader through the complex interplay between bone mineralization and neurological outcomes in preterm infants, this review is structured into several sections. We begin by outlining the pathophysiological mechanisms and risk factors underlying metabolic bone disease of prematurity (MBDP), which lays the foundation for understanding the disorder. Next, we discuss early diagnostic biomarkers and predictive models that facilitate the timely identification of MBDP, including emerging noninvasive techniques such as bioelectrical impedance analysis (BIA). Following this, we examine preventive supplementation strategies with an emphasis on vitamin D dosing and its dual role in enhancing bone health and neurodevelopmental outcomes. Finally, we synthesize these findings in our conclusions and propose future research directions aimed at refining diagnostic and therapeutic protocols.

## 3. Pathophysiology and Risk Factors of MBDP

The basic pathology of MBDP is characterized by inadequate mineralization due to both prenatal deprivation and postnatal nutritional deficiencies [14]. Bone mineralization begins during embryogenesis, with up to 80% of fetal mineral deposition occurring in the last trimester. Premature infants naturally begin life with reduced bone mineral reserves [15]. In this process, osteoblasts secrete the organic bone matrix, creating a scaffold on which calcium and phosphate are deposited, while the concomitant increase in trabecular thickness contributes to an overall increase in bone volume. A shift toward increased osteoclast activity has been documented in genetic or metabolic abnormalities, which disrupts bone remodeling [16].

Before we begin to discuss this disease’s pathological mechanisms, we need to understand the physiological activity of bone mineralization and its relationship to minerals and hormones. Regarding the regulation of calcium (Ca) and phosphorus (P) homeostasis: parathyroid hormone (PTH) secretion is triggered by low serum calcium levels and inhibited by hypomagnesemia and elevated 1,25(OH)_2_D_3_ levels. PTH increases calcium release from bone and renal calcium reabsorption and stimulates renal production of 1,25-dihydroxyvitamin D_3_ (1,25(OH)_2_D_3_). Vitamin D promotes the absorption of calcium and phosphorus from the intestine and facilitates the mobilization of calcium from the bones [9].

**Figure 1:**
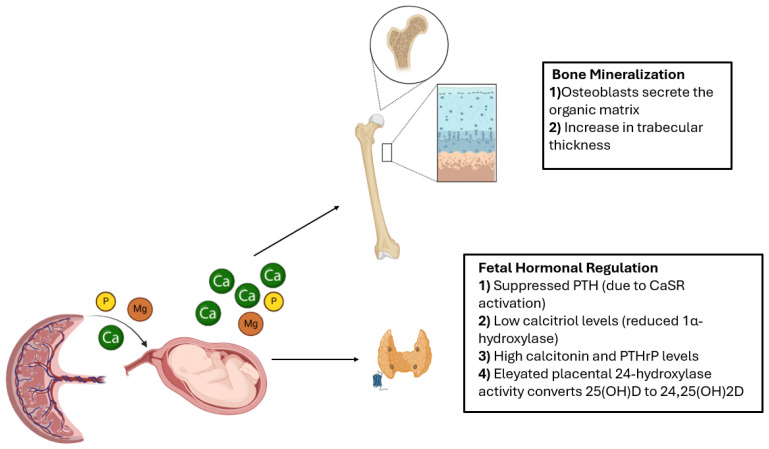
**The prenatal phase of bone mineralization.** Prenatally, the activity of osteoblasts and osteoclasts is heavily dependent on the availability of calcium, phosphate, and magnesium, actively transported by the placenta, which ensure the fetus receives adequate supplies, as the fetal kidneys and gastrointestinal tract contribute minimally to mineral acquisition. Consequently, the fetus presents suppressed parathyroid hormone (PTH), low calcitriol and sex steroid levels, and elevated concentrations of calcitonin and PTH-related protein (PTHrP). Figure created with Biorender.com.

As illustrated in Figure 1, the fetus prenatally exhibits a status of relative hypercalcemia, which is essential for proper skeletal development and is a consequence of the marked placental passage of Ca, P, and magnesium (Mg) [15]. The increase in fetal calcium levels activates the calcium-sensitive receptors (CaSR), which allow for the suppressed secretion of parathyroid hormone (PTH) [17]. Moreover, the reduction in fetal renal 1α-hydroxylase activity as a result of PTH decline, increased calcium and phosphate levels, the influence of fibroblast growth factor-23 (FGF23), and the overactivation of placental 24-hydroxylase leads to decreased calcitriol synthesis. In fact, the role of 24-hydroxylase is to catalyze the hydroxylation of 25-hydroxyvitamin D at the C24 position, yielding 24,25-dihydroxyvitamin D—a catabolic derivative that lacks the structural prerequisites for subsequent 1α-hydroxylation, thereby precluding its conversion into biologically active 1,25-dihydroxyvitamin D (calcitriol) [17]. Increased fetal calcitonin, produced by the thyroid and placenta, and PTHrP, secreted by chondrocytes, perichondrial cells, and osteoblasts, act synergistically to promote mineral storage and regulate fetal calcium and phosphate homeostasis [15]. During fetal life, calcitriol is not essential for maintaining serum mineral levels. Even severe vitamin D deficiency or the absence of a functional vitamin D receptor or 1α-hydroxylase does not markedly affect serum calcium and phosphate concentrations [17]. Nevertheless, clinical studies suggest that vitamin D supplementation during pregnancy may reduce the risk of pre-eclampsia and gestational diabetes, both of which are risk factors for MBDP [18,19]. In addition, genetic polymorphisms affecting the vitamin D receptor have been recognized as predisposing factors for MBDP [20]. Placental mineral transfer mostly peaks in the third trimester, and conditions such as placental insufficiency, intrauterine growth restriction, preeclampsia, and chorioamnionitis increase the risk for MBDP because they can interfere with nutrient transfer [21,22]. The main reason for the susceptibility of preterm infants to MBDP is the fact that the maximum accumulation of calcium and phosphorus in utero occurs mainly around the 34th week of gestation [21,22].

After birth, infants experience sudden shifts in hormonal balance and the disruption of placental mineral transfer associated with respiratory-induced alkalosis, resulting in decreased serum and ionized calcium within the first 24 h [9]. As a result, bone resorption is increased and bone formation is decreased in the first hour of life, with premature infants being particularly susceptible to this process [9].

In the postnatal period, the relationship between calcium, phosphate, and vitamin D intake and the development of MBDP remains controversial [9]. It has been reported that extremely low-birth-weight infants (<30 weeks) who have lower weekly intakes of these nutrients in the first 8 weeks of life are more likely to develop MBDP [8]. Exclusive breastfeeding has been associated with lower phosphate levels than using specialty formula or mineral-enriched milk [19]. Moreover, the incidence of rickets is higher in preterm infants fed unfortified maternal milk (40%) than in those fed specialty formula (16%) [23]. There is also evidence that phosphate supplementation may be more effective in improving biochemical markers of MBDP in preterm infants of lower gestational age and birth weight than in infants with relatively higher levels [24]. Infants with a poor tolerance for enteral feeding who require prolonged total parenteral nutrition (TPN) tend to have suboptimal calcium and phosphate absorption [25].

The last step is, therefore, to know the risk factors associated with MBDP, including lower gestational age, extremely low birth weight, prolonged exposure to parenteral nutrition, and concurrent complications such as necrotizing enterocolitis (NEC), bronchopulmonary dysplasia (BPD), cholestasis, and sepsis [11]. Risk factors for MBDP also concern neurological conditions, in particular, neuromuscular disorders, intraventricular hemorrhage, periventricular leukomalacia, and paralysis [9]. Furthermore, drugs like diuretics, corticosteroids, and anticonvulsants interfere with mineral metabolism [3]. By potentiating osteoclast activity while inhibiting osteoblast function, these conditions promote enhanced calcium resorption and its subsequent renal excretion, ultimately compromising bone deposition [26,27]. For example, bilirubin and bile acids can negatively affect osteoblast function [27]. It is also essential to note that highly elevated bilirubin levels exhibit pronounced neurotoxic effects, as it crosses the blood–brain barrier, thereby precipitating encephalopathy [28].

Finally, inadequate mechanical stimulation, as occurs with reduced fetal movement due to neuromuscular disorders, may also contribute to reduced bone calcification: it represents an example of the association between MBDP and neurological disorders in infants [29]. For this reason, among preventive strategies for MBDP, it is recommended to encourage daily passive mobilization exercises through physiotherapy [30].

## 4. Early Diagnostic Biomarkers and Predictive Models

A major challenge in managing MBDP is the early identification of at-risk infants before irreversible bone loss occurs; in fact, due to its asymptomatic or paucisymptomatic manifestation, this disease is underdiagnosed [5].

Biochemical markers such as serum alkaline phosphatase (ALP) and blood phosphorous levels are widely recognized as early indicators of increased bone turnover. Several studies have demonstrated that elevated ALP levels, particularly in the third week of postnatal age, often rising above 500 IU/L, signal the onset of metabolic derangements [3]. In fact, full-term neonates with ALP >700 U/L present osteopenia with a sensitivity and specificity of 73%, while ALP >800 U/L in preterm infants represents a marker of severe MBDP [31,32]. Lü et al. proposed an ALP cutoff value of 344 U/L as an effective early warning threshold, a value that has been incorporated into diagnostic models with high sensitivity and specificity [33]. Regarding the follow-up timing of ALP, it has been proposed to repeat ALP dosages every 2 weeks [34]. Phosphatemia reduction in MBDP neonates usually manifests around 7–14 days after birth, even if sometimes the reverse regulation of parathyroid hormone (PTH) can cause normal levels of phosphate [35]. In fact, in preterm infants with MBDP, PTH concentrations may be elevated, reflecting a compensatory secondary hyperparathyroidism triggered by the hypocalcemia and/or hypophosphatemia typical of this condition. Nevertheless, a consensus on the reference range of PTH in preterm neonates remains elusive, and PTH concentrations observed in both infants with and without MBDP are comparable [35].

In parallel, emerging data on fibroblast growth factor-23 (FGF23) indicate its potentiality as an early marker. Low FGF23 levels measured at 3–4 weeks of life can predict subclinical hypophosphatemia [35]. MBDP patients showed lower levels of serum phosphorous and FGF23, compared to non-MBDP patients [35].

Risk prediction has also advanced with the development of nomogram models. For example, Liang et al. constructed a nomogram incorporating gestational age, the time of trophic feeding initiation, the duration of parenteral nutrition, and clinical comorbidities (NEC, BPD, cholestasis, and sepsis) [14]. This tool, which achieved an area under the receiver operating characteristic curve (AUROC) of approximately 0.80–0.85, allows clinicians to stratify infants by risk and tailor early interventions accordingly. A nomogram model is valuable because it combines routinely collected clinical variables to provide a visual and user-friendly risk estimation [14].

For neonates at risk for osteopenia, it is essential at 15 days of life to perform serum evaluations of calcium, phosphorus, and alkaline phosphatase (ALP), even if none of these are considered specific markers for MBDP [9,12,30]. If the ALP level is below 500 U/L and serum phosphorus exceeds 5 mg/dL, monitoring should continue at 10–15-day intervals [30]. Conversely, if ALP is above 500 U/L and serum phosphorus is below 5 mg/dL, the tubular reabsorption of phosphorus (TRP) must be calculated from a 24 h urine collection. The formula determining the TRP percentage is as follows:

TRP (%) = [1 − (urinary phosphate/urinary creatinine) × (plasma creatinine/serum phosphate)] × 100.

If the TRP falls within the normal range (78–91%), periodic blood monitoring every 10–15 days is indicated. However, if the TRP exceeds 95%, supplementation should be started [28]. Additional instrumental examinations, when feasible, may include X-ray assessments of the humeri and wrists at eight weeks of life, bone densitometry, and quantitative bone ultrasound evaluations [30].

Notably, the application of bioelectrical impedance analysis (BIA) within NICU settings is increasingly prevalent [36,37,38]. As a rapid, precise, and noninvasive diagnostic tool, it holds promise as an asset in both the detection and screening of neonates at risk for MBDP.

In neonatal care, BIA is increasingly used to estimate body composition and determine fluid balance and nutritional status at the bedside, supporting clinical decision making in the neonatal intensive care setting [39]. The fundamental principle of BIA is based on the fact that tissues with a high water and electrolyte content (such as lean muscle) conduct electrical current more readily than tissues with a lower water content, such as fat or bone [40].

While much of the focus has been on fluid and nutritional monitoring, emerging data hint at BIA’s utility in evaluating skeletal muscle mass. The application of BIA in bone evaluations, specifically through the skeletal muscle mass index, has already been demonstrated in patients receiving intensive care in neurocritical settings [41]. Furthermore, Ito et al. (2024) examined the skeletal muscle mass index in children who were born large for their gestational age and they found that, despite differences in fat accumulation, skeletal muscle mass was similar across groups [42]. These observations imply that BIA-derived fat-free mass measurements might indirectly reflect aspects of musculoskeletal status in neonates, even though direct assessments of bone mineral content remain limited. Applying BIA in this context may prove highly beneficial for clinical decision making, as this swift and minimally invasive assessment can provide early indications of neonates exhibiting osteopenia, thereby identifying those who may require enhanced vitamin and mineral supplementation to prevent bone deterioration. However, as the direct link between BIA and standardized bone mineral density measurements has been described in adults and not extensively corroborated in neonates, further studies are needed to fully investigate BIA’s capacity for skeletal evaluation in newborns.

## 5. Preventive Supplementation Strategies

Early nutritional interventions, particularly the proactive supplementation of calcium and phosphorus, have been shown to favorably modulate biochemical markers of bone metabolism and reduce the incidence of MBDP [4]. A retrospective study of 234 preterm infants (<32 weeks or <1500 g) compared early prophylactic calcium and phosphorus supplementation with a reactive approach (initiated after hypocalcemia or hypophosphatemia) and found that early supplementation significantly improved mineral metabolism [43]. The supplemented group demonstrated significantly higher serum phosphorus and 25-hydroxyvitamin D levels, lower alkaline phosphatase (ALP) and parathyroid hormone levels, and improved growth parameters (weight, length, head circumference, and bone density). Moreover, the incidence of MBDP and associated fractures was significantly reduced in the prophylactic group, highlighting the clinical benefit of early intervention. These findings support the clinical benefit of early mineral supplementation in reducing MBDP in premature infants [43].

Additionally, studies examining vitamin D supplementation regimens, such as those comparing 400 IU versus 600 or 800 IU per day, suggest that higher doses may optimize vitamin D status in extremely preterm infants, indirectly supporting bone mineralization [44,45].

A study protocol regarding a randomized, double-blind trial determining the optimal enteral vitamin D dosage for extremely preterm infants (those born before 28 weeks of gestation or weighing less than 1000 g) is going to compare two supplementation strategies administered within 96 h after birth for the first 28 days: one arm will receive 400 IU/day once feedings have been well established, and the other arm will receive a higher fixed dose of 800 IU/day with any feeding. The main objective is to assess whether the increased dosage raises serum 25-hydroxyvitamin D3 levels by over 33% at one month, while also evaluating its impact on respiratory support needs at 36 weeks postmenstrual age and growth and neurodevelopment over a two-year follow-up [45].

Moreover, in a double-blind trial involving 108 preterm infants (≤32 weeks gestation), two vitamin D regimens (400 IU and 600 IU per day) were compared to prevent osteopenia of prematurity. At five weeks of age, although the higher-dose group exhibited significantly elevated serum 25(OH)D levels, no clear differences emerged between the groups regarding ALP or the clinical and radiographic indicators of rickets [44].

Vitamin D is critical in promoting intestinal calcium and phosphorus absorption; therefore, optimal dosing appears to enhance overall mineral balance [46]. According to ESPGHAN guidelines, nutritional intakes for VLBW neonates should be between 800 and 1000 IU per day, substantially higher than the 400–600 IU per day suggested for term infants, as higher doses have demonstrated greater efficacy [44]. Additionally, calcium requirements are estimated at 120–140 mg/kg per day and phosphorus at 60–90 mg/kg per day [30,46]. In the end, vitamin D should be administered as either ergocalciferol (D2) or cholecalciferol (D3); active metabolites should be reserved for specific clinical conditions. For example, in hepatic insufficiency, 25-hydroxyvitamin D (calcifediol) is recommended, whereas in cases of hypoparathyroidism or renal insufficiency, alfacalcidol or 1-hydroxyvitamin D is advised [30,46].

## 6. Neurological Effects of Osteopenia in Preterm Infants and Preventive Strategies

We focused on the relationship between osteopenia and neurological impairment in newborns. In a population of preterm infants, among several clinical comorbidities for MBDP, neurological conditions showed a statistically significant association; for example, seizures have been reported in 40% of patients affected by MBDP, while none have been reported in patients without MBDP [35].

We assessed whether the specific biochemical alterations characterizing MBDP are associated with concurrent neurological involvement. A marked elevation of ALP levels, with other bone markers within reference limits (vitamin D, P, Ca, and PTH) in the first days of life, has been described in GM1 gangliosidosis, a neurodegenerative disorder with variable skeletal disease [47]. ALP was demonstrated to be an early finding in a small number of cases affected by GM1 gangliosidosis [47,48]. Particularly, GM1 gangliosidosis in infants showed significantly increased ALP levels, compared to late-infantile and juvenile patients [49]. Neurological features in this case are characterized by central hypotonia, head lag, hypertonia in upper and lower limbs, irritability, global developmental delay, and the progressive development of epileptic encephalopathy [50]. These observations confirm the neuronal role of ALP.

Furthermore, as previously noted, neuromuscular disorders represent a significant risk factor for metabolic bone disease in infants [1]. For instance, spinal muscular atrophy (SMA) typically presents in the neonatal period and can now be identified through comprehensive neonatal screening programs [51].

As shown in Table 1, to date, there is a notable gap in the scientific literature on this subject; nevertheless, the association between MBDP and neurological involvement stems from the cascade of neurophysiological disturbances arising from imbalances in the biochemical systems, comprising minerals, vitamins, and hormonal pathways, implicated in osteopenia of premature infants. Moreover, this link is further corroborated by the neuroprotective effects observed with vitamin D and mineral supplementation, as exemplified by studies on vitamin D supplementation [52]. Emerging research highlights a compelling association between inadequate maternal vitamin D levels and suboptimal neurodevelopment in offspring [53]. Low circulating 25(OH)D concentrations during gestation have been linked to diminished cognitive and psychomotor performance in early childhood and may elevate the risk of language disorders and neurodevelopmental disorders, including ADHD and autism spectrum conditions [54,55,56,57]. Particularly, newborns with lower levels of cord blood 25(OH)D demonstrated a higher risk of intellectual disability and delayed psychomotor development, confirming the hypothesis that having a lower neonatal vitamin D status at birth and not only during pregnancy impacts later neurocognitive development [57]. Conversely, supplementation with vitamin D appears to offer neuroprotective benefits, potentially through its anti-inflammatory, immunomodulatory, and antioxidant properties, which may mitigate oxidative stress and support neuronal health. As depicted by Gáll et al., 2021, although interventional trials have produced heterogeneous results, with some studies indicating improved cognitive biomarkers and functional outcomes following supplementation, evidence suggests that maintaining sufficient vitamin D status during pregnancy and beyond could play a critical role in preserving cognitive function [53]. However, methodological inconsistencies in assessing vitamin D status and neurocognitive performance underscore the need for rigorously designed randomized controlled trials to determine optimal dosing strategies.

## 7. Conclusions

To date, this is the first review not only dissecting the pathophysiological mechanisms, diagnostic tools, and preventive strategies for MBDP but also explicitly investigating its connection with neonatal neurological impairment, thereby addressing a critical gap in the existing literature. Additionally, this review underlined the dual role of vitamin D supplementation for bone integrity and neurological outcome improvements.

Taken together, the evidence suggests that a multifaceted approach is needed for the management of MBDP in preterm infants. Early diagnostic measures, centered on biochemical markers such as ALP, calcium, and phosphorus, along with emerging markers like FGF23, and the incorporation of noninvasive tools such as bioelectrical impedance analysis (BIA) in NICU settings, enable clinicians to detect MBDP at a stage when nutritional interventions can still reverse or mitigate bone demineralization. In addition to well-documented skeletal implications, our review highlights a significant yet underrecognized relationship between MBDP and neurological dysfunction in the neonate, in which severe bone demineralization may lead to complications including seizures, central hypotonia, and developmental delays.

Based on current evidence, we propose that routine monitoring should include serum ALP and phosphorus assessments by day 14 of life in preterm neonates (<32 weeks gestation). In neonates with persistent biochemical deficits—specifically, ALP values consistently exceeding 500 IU/L accompanied by low phosphorus levels—an escalation of vitamin D supplementation up to 800 IU/day is recommended. Furthermore, the integration of BIA into routine neonatal evaluations may facilitate the early identification of alterations in body composition associated with MBDP, thereby guiding timely nutritional interventions. These practical proposals offer a preliminary framework to bridge existing research gaps and enhance both skeletal and neurodevelopmental outcomes; however, they warrant validation in future prospective, multicenter studies.

In addition to the well-documented skeletal implications, our review underscores a significant, yet underrecognized, relationship between MBDP and neurological dysfunction in the neonate. Several studies indicate that severe bone demineralization, whether due to genetic conditions or nutritional deficits, can be associated with neurological manifestations such as seizures, central hypotonia, and developmental delays. These disturbances can result in an increased risk of neonatal seizures and subsequent neurodevelopmental impairment. Moreover, preterm infants with neuromuscular disorders are particularly susceptible to both skeletal and neurological complications, emphasizing the need for integrated management strategies, including physiotherapy. Preventive interventions, including early supplementation with calcium, phosphorus, and particularly, vitamin D, have demonstrated improvements in bone metabolism and growth parameters. Optimizing vitamin D status may not only enhance mineral absorption and bone strength, but also confer neuroprotective benefits, likely through anti-inflammatory, immunomodulatory, and antioxidant mechanisms supporting neuronal health.

Despite these advances, several challenges remain. Variability in the optimal dosing and timing of supplementation and the dynamic changes in biochemical markers during the neonatal period complicate the early diagnosis of MBDP; in fact, to date, there is a lack of clear diagnostic criteria for MBDP [9]. Furthermore, multicenter studies are needed to validate the proposed early predictive models, establish age-specific reference ranges for key biomarkers, and clarify the long-term neurological outcomes of MBDP. Ultimately, addressing these gaps will not only improve skeletal health but may also have significant implications for preserving cognitive function and neurodevelopment.

## Figures and Tables

**Table 1:** Summary of included studies focusing on neurological effects of osteopenia in preterm infants and preventive strategies.

Title, Author, Year, Reference	Type of Study	Findings	Neurological Findings
Metabolic Bone Disease in the Preterm Infant: Current State and Future Directions (Rehman & Narchi, 2015) [1]	Review	Highlights the importance of early biochemical and radiological monitoring and nutritional interventions in preterm infants with MBD	Identifies neuromuscular disorders as risk factors that, via prolonged immobilization, contribute to impaired cortical bone growth and subsequent rickets/fractures.
Persistent Elevations of ALP as an Early Indicator of GM1 Gangliosidosis (Menkovic et al., 2025) [47]	Case report	Suggests persistent ALP elevation as early marker for GM1 gangliosidosis, linking skeletal dysplasia with neurodegeneration	Progressive neurodegenerative signs including central hypotonia, head lag, hypertonia, and hearing loss.
Neurodevelopment Outcomes in Very-Low-Birth-Weight Infants with Metabolic Bone Disease at 2 Years of Age (Chen et al., 2024) [13]	Retrospective study	Assessed neurodevelopmental outcomes at 6, 12, and 24 months of corrected age in 749 VLBW infants (<1350 g), comparing those with and without radiographic signs of MBD.	Infants diagnosed with MBD showed significantly lower scores in cognitive, motor, and language domains at 2 years of age, even after adjusting for confounders. Strong association between MBD and neurodevelopmental delay was demonstrated.
The Impact of Vitamin D Supplementation Duration on Early Childhood Developmental Milestones: A retrospective study (Praticò et al., 2024) [52]	Retrospective study	Indicates that extended vitamin D supplementation may confer modest yet significant neurodevelopmental advantages, likely via enhanced skeletal, neuromuscular, and brain function.	The 12-month supplementation group achieved certain motor (e.g., walking), fine motor (object tracking), and language milestones earlier, with higher Griffiths developmental scores at 1 and 2 years.
Role of Vitamin D in Cognitive Dysfunction: New Molecular Concepts and Discrepancies between Animal and Human Findings (Gáll et al., 2021) [53]	Review	Highlights the complex role of vitamin D signaling in the CNS and underscores that maintaining adequate vitamin D is vital for cognitive development, with systemic implications for skeletal health.	Links vitamin D deficiency with cognitive dysfunction, neurodevelopmental disorders (e.g., ADHD, autism, and schizophrenia), and neurodegenerative diseases; discusses VDR expression in key brain regions.
Maternal Vitamin D Status and Infant Outcomes in Rural Vietnam: a prospective cohort study (Hanieh et al., 2014) [54]	Prospective cohort study	Indicates low maternal vitamin D during late pregnancy is associated with impaired language development in infancy, supporting antenatal vitamin D supplementation to improve neurodevelopmental outcomes.	Infants born to vitamin-D-deficient mothers (<37.5 nmol/L) had significantly lower language composite scores and smaller head circumferences at birth, indicating an adverse neurodevelopmental impact.
Neurodevelopmental Effects of Prenatal Vitamin D in Humans: Systematic Review and Meta-analysis (García-Serna et al., 2020) [12]	Systematic review and Meta-analysis	Evaluated prenatal 25(OH)D (maternal and cord blood) as a marker of vitamin D exposure; reinforced the importance of adequate prenatal vitamin D for neurodevelopment.	Higher prenatal 25(OH)D levels were associated with improved cognitive development (borderline significance) and significantly lower risks of ADHD and autism-related traits.
Cord Blood Vitamin D and Neurocognitive Development Are Nonlinearly Related in Toddlers (Zhu et al., 2015) [57]	Prospective follow-up study	Suggests that there is an optimal cord blood vitamin D level (approximately 30–50 nmol/L) for neurocognitive outcomes; both deficient and excessive vitamin D levels may impair development, highlighting the need for further research on optimal thresholds in early life.	Demonstrated an inverted U-shaped relationship between cord blood 25(OH)D concentrations and neurocognitive development: both the lowest and highest quintiles were associated with significantly lower mental (MDI) and psychomotor (PDI) scores compared to mid-range values.

## Abbreviations

The following abbreviations are used in this manuscript:

MBDP	Metabolic Bone Disease of Prematurity
NEC	Necrotizing Enterocolitis
BPD	Bronchopulmonary Dysplasia
NICU	Neonatal Intensive Care Unit
QUS	Quantitative Ultrasound
VLBW	Very Low Birth Weight
ESPGHAN	European Society for Paediatric Gastroenterology, Hepatology, and Nutrition
Ca	Calcium
P	Phosphorus
ALP	Alkaline Phosphatase
PTH	Parathyroid Hormone
FGF23	Fibroblast Growth Factor-23
TRP	Tubular Reabsorption of Phosphate
BIA	Bioelectrical Impedance Analysis
TPN	Total Parenteral Nutrition
SMA	Spinal Muscular Atrophy
BSID	Bayley Scales of Infant Development
MDI	Mental Development Index
PDI	Psychomotor Development Index
VDD	Vitamin D Deficiency
25(OH)D	25-Hydroxyvitamin D
IU	International Units
D2	Ergocalciferol
D3	Cholecalciferol
DPDIA3	Protein Disulfide Isomerase A3
CNS	Central Nervous System
MPS	Mucopolysaccharidosis
DBP	Vitamin D Binding Protein
AUROC	Area Under the Receiver Operating Characteristic Curve
EEG	Electroencephalogram
MRI	Magnetic resonance Imaging
B-ALP	Bone-Specific Alkaline Phosphatase
PTHrP	Parathyroid Hormone-Related Protein
CaSR	Calcium-Sensing Receptor
Mg	Magnesium
